# *biogitflow*: development workflow protocols for bioinformatics pipelines with git and GitLab

**DOI:** 10.12688/f1000research.24714.3

**Published:** 2021-02-19

**Authors:** Choumouss Kamoun, Julien Roméjon, Henri de Soyres, Apolline Gallois, Elodie Girard, Philippe Hupé

**Affiliations:** 1 Institut Curie, Paris, F-75005, France; 2 U900, Inserm, Paris, F-75005, France; 3 PSL Research University, Paris, France; 4 Mines Paris Tech, Fontainebleau, F-77305, France; 5 UMR144, CNRS, Paris, F-75005, France

**Keywords:** development workflow, bioinformatics pipeline, quality management, healthcare

## Abstract

The use of a bioinformatics pipeline as a tool to support diagnostic and theranostic decisions in the healthcare process requires the definition of detailed development workflow guidelines. Therefore, we implemented protocols that describe step-by-step all the command lines and actions that the developers have to follow. Our protocols capitalized on the two powerful and widely used tools git and GitLab, and are based on gitflow, a well-established workflow in the software engineering community. They address two use cases: a *nominal *mode to develop a new feature in the bioinformatics pipeline and a *hotfix *mode to correct a bug that occurred in the production environment. The protocols are available as a comprehensive documentation at https://biogitflow.readthedocs.io and the main concepts, steps and principles are presented in this report.

## Introduction

The importance of best practices for bioinformatics analysis and software have been highlighted by many authors who proposed very valuable guidelines for better reproducibility and traceability ( Georgeson *et al.*, 2019; Hamburg & Mogyorodi, 2019; Noble, 2009; Sandve *et al.*, 2013). However, reproducing an analysis is often a challenge ( Kim *et al.*, 2018). Therefore, guidelines have to be promoted by the computational labs and bioinformatics core facilities to federate the bioinformaticians across common practices for software development. This is especially essential when the bioinformatics pipeline is used in precision medicine to support the diagnostic and theranostic activities. Indeed, many hospitals worldwide use High-Throughput Sequencing in routine clinical practice to guide the therapeutic decision. This new era of genomic medicine is even promoted in healthcare systems at a large scale within several national initiatives, such as France, USA, UK or Australia ( Stark *et al.*, 2019).

This evolution has brought Bioinformatics at the forefront of the healthcare process with the bioinformatics pipeline being a fully integrated component of the clinical decision. Compliance with the healthcare laboratory accreditation standards and regulations is thus required for the development and exploitation of the bioinformatics pipeline. In this context, several authors ( Hume *et al.*, 2019; INCa, 2018; Matthijs *et al.*, 2016; Roy *et al.*, 2018) recommended in their guidelines that an appropriate code repository tool should be used to enforce version control. It ensures to track the different releases of the bioinformatics pipeline, their validation and the developers involved in their implementation. This must be integrated in a quality management process with standardized protocols approved for a diagnostic use.

There is a wide ecosystem of tools (see Perez-Riverol *et al.*, 2016; Riesch *et al.*, 2020; Zolkifli *et al.*, 2018) that can support the implementation of such protocols. Among them, we capitalized on git for the version-control system as it became a very popular tool in the software engineering community and GitLab for the repository manager as it can be self-hosted. Both tools offer a large set of very powerful functionalities that can be used and combined in multiple ways thus increasing their usage complexity. It is thus mandatory to formalize through detailed guidelines how to use them on a daily basis for the development and deployment of the bioinformatics pipeline. Therefore, we implemented the set of protocols *biogitflow* ( Kamoun *et al.*, 2020) that describes step-by-step all the command lines and actions to be performed by the developers.

These protocols are mainly dedicated to bioinformaticians and software developers who are involved in the development, deployment and maintenance of bioinformatics pipelines that support routine production. For example, core facilities (such as sequencing platforms) generate, with a very a high-throughput, many data that have to be processed and delivered on the fly to the end-users. Short time-to-delivery and continuity of service under any circumstances is a challenge that can be tackled by promoting these protocols to pave the way towards better industrialized processes for the software component in the context of production, in particular in healthcare.

A comprehensive documentation available at https://biogitflow.readthedocs.io provides all the technical details. We introduce here the main concepts, steps and principles.

## Methods

### Development workflow

The development workflow consists of four main steps. The first step is **software development**, which includes code writing and testing. The second step is **acceptance testing** by the end-users who validate that the expected functionalities have been correctly implemented. The third step is **check the installation process and new testing** to ensure that the bioinformatics pipeline can be installed in a similar environment than the one used in production. During this step, a new testing is performed such that bugs can be corrected before installing the bioinformatics pipeline in production. Finally, the fourth step is **production deployment**. During this last step, the new release of the bioinformatics pipeline with the new functionalities is installed in the production environment.

### Multiple deployment environments

The four different steps have to be performed in separated environments in order to i) ensure that a stable version can be used in production, ii) allow the end-users to validate a new release without any impact on both the version used in production or the version under development and iii) allow the software developers to add new functionalities and modify the code without any impact on the end-users who are validating a new version and/or using the current version in production. Therefore, three deployment environments are used: a development ( **dev**), a validation for pre-production ( **valid**) and a production environment ( **prod**). Besides these environments, each developer can deploy the bioinformatics pipeline in a **local** workspace to test the new functionalities that have been developed.

### Version control and branching model

Several development workflows and branching models have been proposed including GitHub flow, GitLab Flow and gitflow. Our *biogitflow* protocols capitalized on the popular gitflow model proposed by Driessen (2010) ten years ago. The management of the different bioinformatics pipeline versions is based on four different git branches. Depending on the context and the step of the development workflow the following branches on the remote repository are used:

**devel** contains the code of the current version under development.

**release** contains the code with both candidate and official releases. The release branch comes from the devel branch.

**hotfix** is a mirror of the release branch and is used to patch the code that is in production. If a critical bug occurs in production, this branch is used to fix the issue.

**master** is only used to archive the code from the release and hotfix branches. This branch is not used for development.

Among these four branches, the release, hotfix and master are protected branches such that only the developers with the Maintainer role in the GitLab repository can directly push code on these branches (the other users have to use the GitLab merge request functionality to push on protected branches).

In addition to these four branches, the developer can create local branches to i) implement a new feature (the branch is named with a prefix feature plus any meaningful suffix) and ii) fix a bug in production or resolve a problem during the third step (these branches are either named with the prefix release or hotfix depending on the use case plus some relevant contextual information).

### User roles and permissions

Two levels of roles and permissions are considered. Firstly, in the GitLab remote repository a developer is either assigned to the role Developer ( **D**) to push the developments only on the non-protected branches or Maintainer ( **M**) to push the developments on any branches. Secondly, in the deployment environments a user is either granted with the permission **UD** to deploy in the dev environment only or **UVP** to deploy in the valid and prod environments (any user with the **UVP** permissions is also granted with the **UD** permissions).

### Bioinformatics pipeline testing

Testing the bioinformatics pipeline occurs during all the steps of the development workflow. It involves all stakeholders including the developers, the users in charge of the deployments and the end-users. This should be done as often as possible to identify and resolve any unexpected behavior early in the development process. The International Software Testing Qualifications Board provides comprehensive guidelines for software testing ( Hamburg & Mogyorodi, 2019). Among them, we strongly recommend to include the following testing. The *unit testing* confirms that a piece of code provides the expected output according to the input parameters. The *integration testing* checks that the interfaces of the different bioinformatics pipeline components are consistent with each other and that the result of their integration allows the expected functionalities to be performed. The *system (or functional) testing* validates that the full bioinformatics pipeline works and fits well the end-user’s needs. The *regression testing* checks that the correction of bugs or the development of new functionalities did not introduce defects in unchanged areas of the bioinformatics pipeline. In addition, we highlight the importance of the *operational testing* to check that the bioinformatics pipeline provides the expected results on a reference dataset (golden dataset) in the production environment. This testing is systematically launched prior to any new analysis by the bioinformatics pipeline in the production environment. This ensures that the results are reproducible as long as the exact same version of the bioinformatics pipeline is used.

## Results

**Figure 1.**
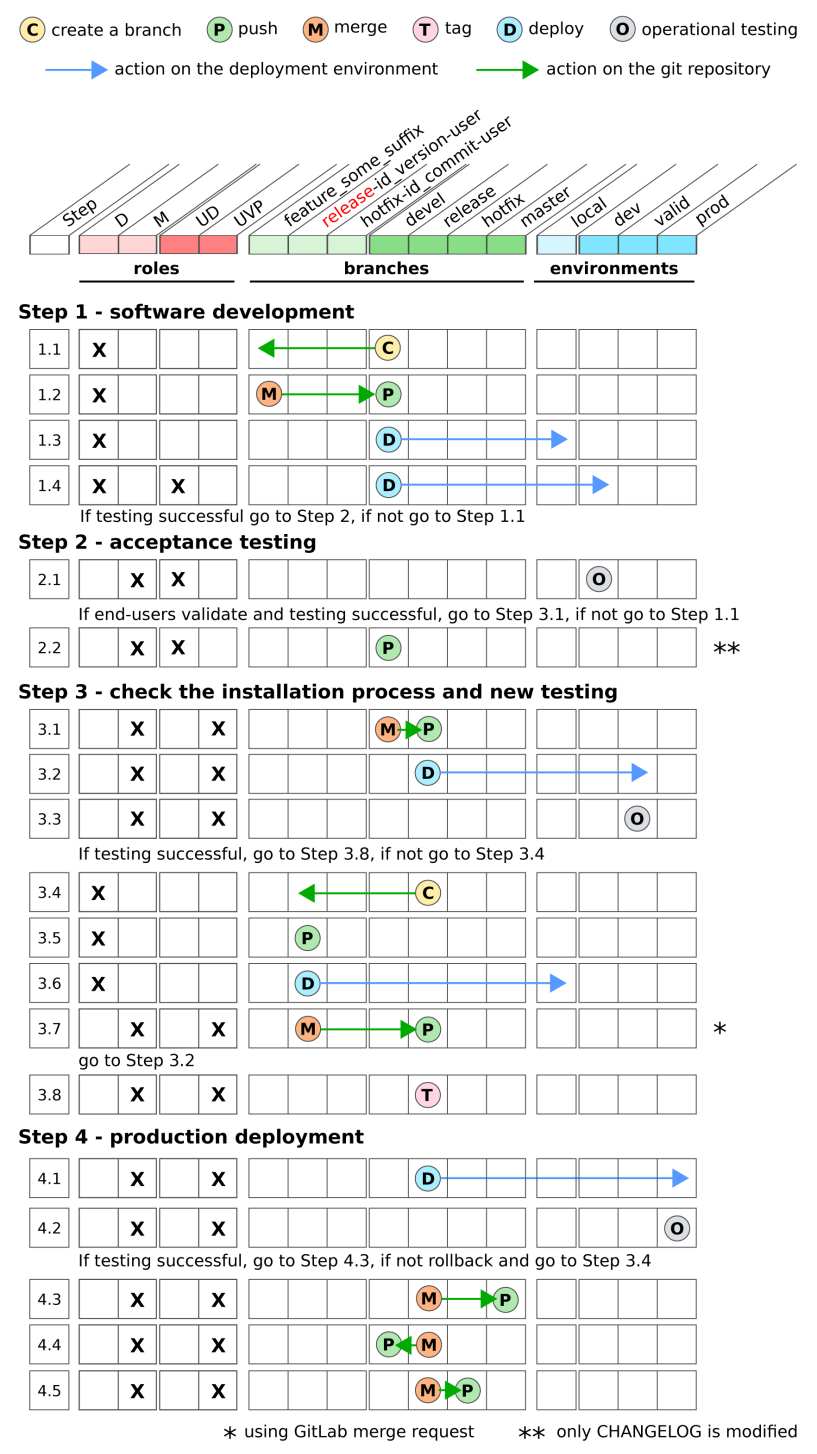
*biogitflow* protocol for the nominal mode. This graphical synopsis provides an overview of the different steps of the development workflow when a new feature is implemented in the bioinformatics pipeline according to the role and permissions of the developer. It describes the different git actions that are performed on the different branches and the deployments in the different environments.

**Figure 2.**
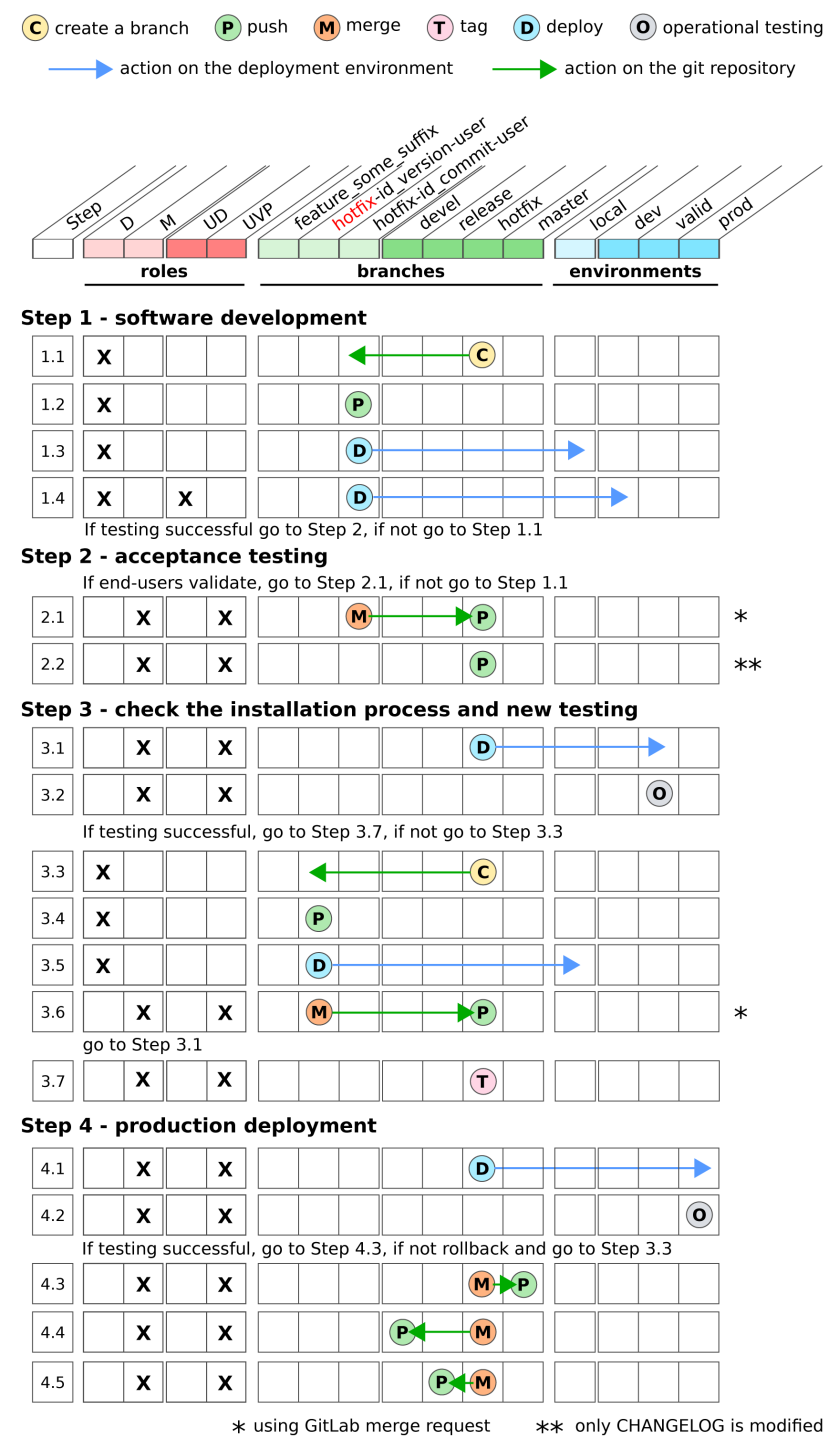
*biogitflow* protocol for the hotfix mode. This graphical synopsis provides an overview of the different steps of the development workflow when a bug occurred in the production environment in the bioinformatics pipeline according to the role and permissions of the developer. It describes the different git actions that are performed on the different branches and the deployments in the different environments.

Two use cases have been addressed with dedicated protocols that detail the different actions step-by-step. The first one is the **nominal mode** ( Figure 1) in which a new feature is implemented to improve the bioinformatics pipeline based on requirements and expectations from the end-users who operate it for their daily clinical practice. The second one is the **hotfix mode** ( Figure 2) in which there is a bug in the bioinformatics pipeline in the production environment that hampers the delivery of the results for the patients. The *biogitflow* documentation provides all the technical details, on how to configure the remote repository in GitLab to develop a new bioinformatics pipeline, how to use git and GitLab depending on the roles and permissions during the time frame of the development workflow for both use cases.

Whatever the use case, the bioinformatics pipeline is deployed in production and operated by the end-users once it has successfully passed all the testing. Whenever new patient data has to be analyzed to deliver results for diagnostic or theranostic purposes, the bioinformatics pipeline can be launched by the end-users only if the *operational testing* reproduces the results from the golden dataset, otherwise it is blocked by internal control mechanism. In this case, the developers investigate the reason of the failure and have to fix it using the **hotfix mode**.

As a real example, we provided a repository that is available at https://gitlab.com/biogitflow/biogitflow-template (it is recommended that the user registers on GitLab.com to benefit from all its functionalities). This repository has been created according to the guidelines from the section *Create a new project in Gitlab* from the *biogitflow* documentation. It contains the different protected and non-protected branches according to the branching model, protected tags, templates for the issues and merge requests, and a set of labels for the issues. The user can fork the repository in a personal workspace and apply the **nominal mode** ( Figure 1) and **hotfix mode** ( Figure 2) following the proposed protocols.

## Discussion

While gitflow was a main source of inspiration, *biogitflow* differs in several ways as:

- it uses a similar branching model with some adaptations:
  - gitflow relies on two branches with infinite lifetime, the master and devel(op) branches, and three branches with limited lifetime, the feature (a local branch on the developer’s side), release and hotfix branches that could be eventually removed from the remote repository. In contrast, the release and hotfix branches have infinite lifetime in *biogitflow*. This way, the release branch is always ready to welcome the future version. The infinite lifetime of the hotfix branch is motivated to address the following situation: the release branch has been modified since a new version is under preparation but a bug occurs in the production environment. Therefore, the hotfix branch is the only branch that is similar to what has been deployed in production and it can be used straightforwardly to fix the bug.
  - the release branch in *biogitflow* is used in a similar manner as the master branch in gitflow, in particular, the tag for a new version is added on the release branch (or hotfix branch in the hotfix mode).
  - the master branch in *biogitflow* is used as an archive of all the developments.
- it provides more technical details about the git commands including the use of commits (with some naming conventions for the commit messages, the tagging).
- it describes the usage of git with GitLab and therefore describes the use of issues, labels and merge requests.
- it is more comprehensive as it actually goes beyond the usage of git and GitLab since it includes guidelines for deployment and testing.
- it defines roles and permissions for the branches and deployment environment.

Writing these protocols required some feedback from the developers in order to decide what was the best way to capitalize on git and GitLab taking into account our internal constraints and organization. The need of formalization required by the laboratory accreditation agency such that a bioinformatics pipeline can be used in healthcare was a great catalyst to implement these protocols. It was a major step to improve our daily practice of software engineering towards better quality management and was a source of motivation to change our work habits. More precisely, the use of the *biogitflow* protocols contributed to the harmonization of the development practices across developers. Every git pipeline repository is now structured the same way that simplifies the skill transfer from one person to another. This is very convenient, especially when you have to maintain different pipeline repositories with different developers involved. Moreover, due to multiple deployment environments along with the branching model, the developer can really perceives a constructive and positive pressure as long as the deployment in the production (the danger zone) is getting close: this is a source of stimulation for better quality.

In order to promote these protocols, it was necessary to train the users involved in the development workflow to demonstrate to the laboratory accreditation agency that the technical protocols are known, understood and mastered by the developers. Therefore, an internal accreditation process of our developers was implemented. The training consists of a series of exercises that cover all the different use cases, roles and permissions of the protocols. The exercises are first realized in pair with the tutor and the trainee, then the trainee performs the exercises alone twice, and finally the trainee performs the exercises alone but in the presence of the tutor who ask additional questions to ensure that the tricky parts of the protocols are understood. To be endorsed, the trainee has to perform the exercises fluently in full autonomy. All the actions of the training process are tracked in a dedicated GitLab remote repository. Endorsement is valid for one year that can be extended if the developer still masters the protocols. This is assessed during a dedicated yearly interview with the tutor. The exercises are available on https://biogitflow.readthedocs.io.

As depicted in Figure 1 and Figure 2, the development workflow protocols contains many steps that are complex. This complexity arises from the reality of the numerous tasks to be performed to ensure the development, deployment and maintenance of bioinformatics pipelines in the context of production as mentioned in the introduction. Writing the documentation was clearly mandatory in order to provide a formal description of our work methods and promote them internally. Obviously, such protocols contains many manual operations that are prone to error and misapplication. This is the reason why, not only training is important, but also practicing on a weekly (or at least on a monthly) basis is crucial to remain fluent. To circumvent these pitfalls, these protocols would benefit from improved automation to reduce the number of manual steps and avoid possible mistakes. This will be the scope of further improvements that could capitalize of additional valuable functionalities such as git hooks, GitLab *CI/CD* (continuous integration / continuous deployment) and *Operations* features.

## Conclusions

We described two protocols that are used on a daily practice to develop bioinformatics pipelines compliant with the accreditation standards for healthcare. Bioinformaticians and software developers involved in the development, deployment and maintenance of bioinformatics pipelines in the context of production will benefit from these protocols. While some choices were made to match our internal constraints and organization, the protocols can be easily transposed in other institutes as the main concepts, steps and principles hold in most of the contexts. Indeed, the protocols were internally motivated by the development of bioinformatics pipelines to support the use of genomics sequencing in clinical diagnostic and can be straightforwardly applied not only to genomics but also to any kind of omics approaches (proteomics, metabolomics, etc.), medical informatics or more generally any software development whether it is bioinformatics or not. According to the principle of the Deming wheel of continuous improvement in quality management ( Swamidass, 2000), the protocols we described are intended to evolve in order to address future requirements, handle new risk management, integrate new tools or technical frameworks offering better efficiency of the overall process. We strongly encourage the promotion of such protocols in the context of production not only for the healthcare activities but also for research activities.

## Data availability

### Underlying data

All data underlying the results are available as part of the article and no additional source data are required.

### Extended data

***biogitflow* documentation is available at:** https://biogitflow.readthedocs.io/.

**Archived source of the *biogitflow* documentation at time of publication:** https://doi.org/10.5281/zenodo.4537153

**License:** CeCILL Version 2.1.

## Author contributions

CK, HDS, JR and PH conceived the protocols. PH wrote the protocols and the manuscript that were reviewed by AG, CK, EG, HDS and JR. PH supervised the study.
