# Supplementary material for: biogitflow: development workflow protocols for bioinformatics pipelines with git and GitLab
Source: F1000Res. 2021 Feb 19;9:632. Originally published 2020 Jun 22. [Version 3] doi: 10.12688/f1000research.24714.3 (PMC7921891; doi:10.12688/f1000research.24714.3)
Supplement: Peer review history

# Peer review: Reviewer response for version 3

**DOI:** 10.5256/f1000research.54697.r79930

**Published:** 2021-03-01

**Authors:** Bernard Pope

**Affiliations:** 1 Melbourne Bioinformatics, University of Melbourne, Parkville, Vic, Australia

I am happy that the authors have addressed all of my comments and suggestions from the previous version.

 A few small typos remain and the article would benefit from a thorough proof read, but they are only minor issues.

Is the work clearly and accurately presented and does it cite the current literature?

Yes

If applicable, is the statistical analysis and its interpretation appropriate?

Not applicable

Are all the source data underlying the results available to ensure full reproducibility?

Yes

Is the study design appropriate and is the work technically sound?

Yes

Are the conclusions drawn adequately supported by the results?

Partly

Are sufficient details of methods and analysis provided to allow replication by others?

Yes

Reviewer Expertise:

Bioinformatics. Genomics. Cancer. Software Development. Pipelines.

I confirm that I have read this submission and believe that I have an appropriate level of expertise to confirm that it is of an acceptable scientific standard.

# Peer review: Reviewer response for version 2

**DOI:** 10.5256/f1000research.31091.r76180

**Published:** 2021-01-21

**Authors:** Julia Ponomarenko

**Affiliations:** 1 Center for Genomic Regulation (CRG), The Barcelona Institute of Science and Technology, Barcelona, Spain

This paper presents the protocol for the development and support of bioinformatics pipelines using git and GitLab. The intended users are bioinformaticians working in a clinical setting, while bioinformaticians working in research/academia can also benefit.

 This reviewer questions if the scope of the intended audience can be expanded. For example, in a clinical setting, software developed in the field of medical informatics is often used along with the software developed in biology. Would the medical informatics benefit from the proposed approach as well? Also, the first paragraph mentions only genomics sequencing as a field of application. But could the same approach at the workflow development be applied to proteomics, metabolomics and other relevant for personalized medicine and diagnostics fields?

**Minor comment:**

- It shouldn’t belie the value of the presented protocol, but the reader and potential user might benefit and become more enthusiastic about using the *biogitflow* if the presented resource provided and described an example of a specific pipeline with a small test dataset.
- The paper requires proofreading for typos; e.g., “As a real example, we provided a of the repository that is available …”

Is the work clearly and accurately presented and does it cite the current literature?

Yes

If applicable, is the statistical analysis and its interpretation appropriate?

Not applicable

Are all the source data underlying the results available to ensure full reproducibility?

Partly

Is the study design appropriate and is the work technically sound?

Yes

Are the conclusions drawn adequately supported by the results?

Partly

Are sufficient details of methods and analysis provided to allow replication by others?

Partly

Reviewer Expertise:

bioinformatics

I confirm that I have read this submission and believe that I have an appropriate level of expertise to confirm that it is of an acceptable scientific standard.

# Response

**Published:** 2021-02-12

**Authors:** Philippe Hupé

**Affiliations:** Institut Curie, France

First, we would like to thank the reviewer. We are very grateful for her time, contribution and very valuable comments that significantly helped to improve the article.

 You will find below a detailed answer to the different issues that we have addressed in the revised manuscript.

 Best regards,

 Choumouss Kamoun, Julien Roméjon and Philippe Hupé

 === Detailed response to the reviewer2's comments ===

 > This reviewer questions if the scope of the intended audience can be expanded. For example, in a clinical setting, software developed in the field of medical informatics is often used along with the software developed in biology. Would the medical informatics benefit from the proposed approach as well? Also, the first paragraph mentions only genomics sequencing as a field of application. But could the same approach at the workflow development be applied to proteomics, metabolomics and other relevant for personalized medicine and diagnostics fields?

 This is really true that the proposed protocols are not restricted to genomics.

 We have added in the conclusion that they can be applied straightforwardly to

 any omics bioinformatics pipelines and even any software development.

 > It shouldn’t belie the value of the presented protocol, but the reader and potential user might benefit and become more enthusiastic about using the biogitflow if the presented resource provided and described an example of a specific pipeline with a small test dataset.

 The biogitflow protocols come with a biogitflow-template that is publicly

 available on GitLab.com. In this new version, we have added in the biogitflow

 documentation an 'Exercises' section that first explains how the user can fork

 the GitLab repository. Then, the user can practice the different exercises. As

 biogitflow is mainly dedicated to the use git and GitLab and not the coding

 standard or the code itself, it is difficult to provide a specific pipeline

 with a test dataset. Therefore, we strongly encourage the user to start from

 the template and go through the different exercises just following the

 technical protocols available on the documentation.

 > The paper requires proofreading for typos; e.g., “As a real example, we provided a of the repository that is available …”

 Typos have been fixed.

# Peer review: Reviewer response for version 2

**DOI:** 10.5256/f1000research.31091.r75901

**Published:** 2021-01-19

**Authors:** Bernard Pope

**Affiliations:** 1 Melbourne Bioinformatics, University of Melbourne, Parkville, Vic, Australia

I thank the authors for their careful consideration of the issues raised in the previous review. I believe the changes made to the paper in response to those issues have improved the manuscript. I believe the following, relatively minor, issues should be considered in the next revision of the paper:

- The abstract should mention that biogitflow is based on gitflow, a well-established workflow in the software engineering community.
- It is not clear what "for any registered user" means, please clarify.
- Typo: "remoterepository", should be "remote repository".
- Some of the key differences between gitflow and biogitflow are enumerated, but additional explanation as to why they are different would be useful. For example, "In contrast, the release and hotfix branches have infinite lifetime in biogitflow," would benefit from a short explanation as to why this is the case.
- "that makes easy" -> "that simplifies"?
- "make the developer feels" (rephrase).
- "As a corollary of these protocols was the necessity" (rephrase).
- Are the training materials used in the accreditation process publicly available? It was not clear if they are included in the biogitflow documentation. If they are available then perhaps a link could be provided. If they are not available then perhaps the authors could consider including them in the documentation.
- If possible, it would be useful to provide a citation for the "Deming wheel of continuous improvement in quality management".

Is the work clearly and accurately presented and does it cite the current literature?

Yes

If applicable, is the statistical analysis and its interpretation appropriate?

Not applicable

Are all the source data underlying the results available to ensure full reproducibility?

Yes

Is the study design appropriate and is the work technically sound?

Yes

Are the conclusions drawn adequately supported by the results?

Partly

Are sufficient details of methods and analysis provided to allow replication by others?

Yes

Reviewer Expertise:

Bioinformatics. Genomics. Cancer. Software Development. Pipelines.

I confirm that I have read this submission and believe that I have an appropriate level of expertise to confirm that it is of an acceptable scientific standard, however I have significant reservations, as outlined above.

# Response

**Published:** 2021-02-12

**Authors:** Philippe Hupé

**Affiliations:** Institut Curie, France

First, we would like to thank the reviewer. We are very grateful for his time, contribution and very valuable comments that significantly helped to improve the article.

 You will find below a detailed answer to the different issues that we have addressed in the revised manuscript.

 Best regards,

 Choumouss Kamoun, Julien Roméjon and Philippe Hupé

 === Detailed response to the reviewer1's comments ===

 > The abstract should mention that biogitflow is based on gitflow, a well-established workflow in the software engineering community.

 We have modified the abstract accordingly

 > It is not clear what "for any registered user" means, please clarify.

 We have rephrased and explained that it is recommended that the user registers

 on GitLab.com

 > Typo: "remoterepository", should be "remote repository".

 The typo was indeed present in the online pdf but not in the word file we used

 to submit the revised version. I guess the editor can fix the typo.

 > Some of the key differences between gitflow and biogitflow are enumerated, but additional explanation as to why they are different would be useful. For example, "In contrast, the release and hotfix branches have infinite lifetime in biogitflow," would benefit from a short explanation as to why this is the case.

 Indeed, more explanation was required. We have added few lines in the

 corresponding paragraph to motivate the infinite lifetime of the release and

 hotfix branch.

 > "that makes easy" -> "that simplifies"?

 We have modified the text.

 > "make the developer feels" (rephrase).

 We have rephrased the text.

 > "As a corollary of these protocols was the necessity" (rephrase).

 >  

 > Are the training materials used in the accreditation process publicly available? It was not clear if they are included in the biogitflow documentation. If they are available then perhaps a link could be provided. If they are not available then perhaps the authors could consider including them in the documentation.

 The exercises were not initially included. We have mentioned in the article

 that they are now available in the version-1.1.0 of the biogitflow documentation.

 > If possible, it would be useful to provide a citation for the "Deming wheel of continuous improvement in quality management".

 The reference to Swamidass (2000) has been added.

# Peer review: Reviewer response for version 1

**DOI:** 10.5256/f1000research.27261.r72478

**Published:** 2020-10-22

**Authors:** Bernard Pope

**Affiliations:** 1 Melbourne Bioinformatics, University of Melbourne, Parkville, Vic, Australia

Motivated by laboratory accreditation requirements for the development of bioinformatics workflows related to high throughput sequencing in a clinical setting, the authors have developed a software development process formalisation based on gitflow, a popular model in mainstream software engineering practice.

 The formalisation prescribes a branching, testing, merging and release process, with set roles and permissions for developers, and methods for managing the development of new features (nominal mode) and bug fixing (hotfix mode).

 In addition to describing the biogitflow workflow, the paper also contains a short section on testing bioinformatics software pipelines, however, this is largely a recapitulation of standard testing terminology, and does not appear to be particularly bioinformatics-specific.

 The paper has a brief discussion about the motivation for adopting the workflow and some unsubstantiated claims about improving their software engineering practices. It also has a brief discussion about the need to train developers in the workflow and formal accreditation.

 Two large figures are included in the paper that attempt to illustrate the proposed workflow in nominal and hotfix modes.

 While the topic of this paper is interesting and highly relevant to bioinformatics software developers working in areas of clinical application, where process accreditation is a key concern, there are a number of areas where I think the paper could be improved:

1. The current version of the paper is lacking in critical analysis of the proposed workflow. One does not have to look far to find criticism (positive and negative) of gitflow. However, this paper does not provide any significant discussion of the pros and cons of the approach. One of the main criticisms of gitflow is that it is overly complex (for some software development projects) and thus prone to error and misapplication. This complexity is illustrated in Figures 1 and 2 and the discussion in the paper about the need to train developers in its use. Given the apparent regimented nature of the process, it seems plausible that some of the complexity could be avoided by automation. Are there any useful tools that can help developers apply the model successfully and avoid the common criticisms of gitflow? If developers are finding it difficult to follow, especially the "tricky" parts then that may be a sign of limitations in the process or limitations in the tools supporting its application.
2. It is not clear how significantly biogitflow differs from gitflow. In what sense does the proposed workflow differ from established software engineering practices?
3. What alternative competing development models exist?
4. How can the authors be sure that the workflow is in fact making a positive difference to their software development?
5. It would be useful to clarify the intended audience of the paper. How can they apply and benefit from this work? 
6. The discussion of the provided biogitflow templates sounds useful, but the discussion in the paper is unclear. Perhaps this could be expanded upon?
7. The paper ends with "We strongly encourage the promotion of such protocols not only for the healthcare activities but also for research activities." However, due to the aforementioned lack of critical analysis, it is not clear from the paper why this is true. 

Is the work clearly and accurately presented and does it cite the current literature?

Yes

If applicable, is the statistical analysis and its interpretation appropriate?

Not applicable

Are all the source data underlying the results available to ensure full reproducibility?

Yes

Is the study design appropriate and is the work technically sound?

Yes

Are the conclusions drawn adequately supported by the results?

Partly

Are sufficient details of methods and analysis provided to allow replication by others?

Yes

Reviewer Expertise:

Bioinformatics. Genomics. Cancer. Software Development. Pipelines.

I confirm that I have read this submission and believe that I have an appropriate level of expertise to confirm that it is of an acceptable scientific standard, however I have significant reservations, as outlined above.

# Response

**Published:** 2020-11-27

**Authors:** Philippe Hupé

**Affiliations:** Institut Curie, France

First, we would like to thank the reviewer. We are very grateful for his time, contribution and very valuable comments that significantly helped to improve the article.

 You will find below a detailed answer to the different issues that we have addressed in the revised manuscript.

 Best regards,

 Choumouss Kamoun, Julien Roméjon and Philippe Hupé

**Detailed response to the reviewer1's comments**

*> Motivated by laboratory accreditation requirements for the development of bioinformatics workflows related to high throughput sequencing in a clinical setting, the authors have developed a software development process formalisation based on gitflow, a popular model in mainstream software engineering practice.*

**

*> The formalisation prescribes a branching, testing, merging and release process, with set roles and permissions for developers, and methods for managing the development of new features (nominal mode) and bug fixing (hotfix mode).*

**

*> In addition to describing the biogitflow workflow, the paper also contains a short section on testing bioinformatics software pipelines, however, this is largely a recapitulation of standard testing terminology, and does not appear to be particularly bioinformatics-specific.*

**

*> The paper has a brief discussion about the motivation for adopting the workflow and some unsubstantiated claims about improving their software engineering practices. It also has a brief discussion about the need to train developers in the workflow and formal accreditation.*

**

*> Two large figures are included in the paper that attempt to illustrate the proposed workflow in nominal and hotfix modes.*

**

*> While the topic of this paper is interesting and highly relevant to bioinformatics software developers working in areas of clinical application, where process accreditation is a key concern, there are a number of areas where I think the paper could be improved:*

**

*> 1. The current version of the paper is lacking in critical analysis of the proposed workflow. One does not have to look far to find criticism (positive and negative) of gitflow. However, this paper does not provide any significant discussion of the pros and cons of the approach. One of the main criticisms of gitflow is that it is overly complex (for some software development projects) and thus prone to error and misapplication. This complexity is illustrated in Figures 1 and 2 and the discussion in the paper about the need to train developers in its use. Given the apparent regimented nature of the process, it seems plausible that some of the complexity could be avoided by automation. Are there any useful tools that can help developers apply the model successfully and avoid the common criticisms of gitflow? If developers are finding it difficult to follow, especially the "tricky" parts then that may be a sign of limitations in the process or limitations in the tools supporting its application.*

 We fully agree that the protocols are complex and it is not straightforward to master them. Of note, we pointed out in our reply to you comment number 5 that the protocols are mainly relevant in the context of production to deliver to process data on the fly which is obviously a challenge especially when the bioinformatics pipelines have to deal with a very high-throughput of data. The complexity of the workflow just reflects the reality of the development and deployment cycle in the context of production as we intend to promote industrialized processes for the software implementation in order to offer better service, better reactivity, better traceability to end-users. Formalism was thus required and this is the reason why we wrote the biogitflow documentation.

 You are also fully right when you say that it is "prone to error and misapplication" and that some "complexity could be avoided by automation". This is clearly an improvement according the Deming wheel as the next step would be to capitalize on additional functionalities of git/gitlab using git hooks or gitlab-CI/CD (continuous integration, continuous deployment) and gitlab Operations features for example.

 Anyway, as you have mentioned, this critical analysis and limitations were absolutely not highlighted. We, therefore, added a new paragraph at the end of the discussion to explain limitations and foreseen improvements with automation.

*> 2. It is not clear how significantly biogitflow differs from gitflow. In what sense does the proposed workflow differ from established software engineering practices?*

 This comparison was clearly missing. We added a new paragraph at the beginning of the discussion that explains the main differences.

*> 3. What alternative competing development models exist?*

 Other alternatives exist such as GitHub flow and GitLab flow. We added them in the section "Version control and branching model".

*> 4. How can the authors be sure that the workflow is in fact making a positive difference to their software development?*

 This is a good question that is difficult to answer. This would require something like an experimental design comparing two or more workflows based on the assessment of objective performance metrics, and then compare the metrics to figure out if they are statistically different. We did not perform such an approach.

 However, we can say that the benefit of the biogitflow protocols was two-fold: 1/ "harmonization of the development practices" and 2/ the "constructive and positive pressure" for "better quality".These points have been added at the end of the second paragraph of the discussion.

*> 5. It would be useful to clarify the intended audience of the paper. How can they apply and benefit from this work?*

 Indeed, the target audience was not clearly mentioned. Therefore, we added in the introduction that the protocols are appropriate for software developers  in the "context of production" to "support core facilities" that generate "high-throughput of data". The protocols aim at promoting better "industrialized processes". The "context of production" has been reminded in the conclusion as well.

 Obviously, any bioinformatician involves in a custom development to analyze data in a research project would not straightforwardly benefit from our work : for this use case, it might just be sufficient to track the developments with git using a single branch. However, we encourage anyone to read the proposed protocols for general knowledge as it can also give ideas for more elaborated needs.

*> 6. The discussion of the provided biogitflow templates sounds useful, but the discussion in the paper is unclear. Perhaps this could be expanded upon?*

 We realized that both the link to the template and its content were indeed missing. We, therefore, added the url to the template and a more detailed description that also refers to the dedicated section in the biogitflow documentation website.

*> 7. The paper ends with "We strongly encourage the promotion of such protocols not only for the healthcare activities but also for research activities." However, due to the aforementioned lack of critical analysis, it is not clear from the paper why this is true.*

 As mentioned in point number 5, we have detailed in the introduction who was the targeted audience and highlighted that these protocols are useful in the "context of production". We have modified the quoted sentence above highlighting again in the conclusion the usefulness of the protocols in the "context of production".
